# The ACAMTO study—impact of add-on osteopathic treatment on adolescent patients with anorexia nervosa: study protocol for a randomized controlled trial

**DOI:** 10.1186/s13063-021-05810-8

**Published:** 2021-11-24

**Authors:** Aurélie Letranchant, Yunkyung Kim-de Montebello, Corinne Dugré-Le bigre, Agathe Wagner, Florence Curt, Jérôme Silva, Isabelle Nicolas, Pablo Votadoro, Nina Kalindjian, Anna Korchonnoff, Andréa Gutierre, Ana Beatriz Novelli, Alexandra Pham-Scottez, Maurice Corcos

**Affiliations:** 1 Adolescent and Young adult psychiatry Unit, Institut Mutualiste Montsouris, 42 boulevard Jourdan, 75014 Paris, France; 2 Osteopath, 101 rue de Javel, 75015 Paris, France; 3 Department of Research, CEESO, 175 Boulevard Anatole France, 93200 Saint-Denis, France; 4 Doctoral school « Recherches en psychanalyse et psychopathologie (ED 450) », Paris University, Paris, France; 5 DOMUS, GHU Paris Psychiatry & Neurosciences, Paris, France

**Keywords:** Anorexia nervosa, Body approach, Interoception, Osteopathic medicine, Osteopathic treatment, Randomized controlled trial

## Abstract

**Background:**

Anorexia nervosa (AN) mainly affects women (sex ratio 1/10) and most often starts during adolescence. The prognosis of AN remains poor (10% of deaths and high risk of chronicity). Body dissatisfaction, disturbances in recognition and identification of body sensations are some of the key symptoms of AN. However, there is a contrast between this consensual observation of body image disorders in AN, and the relative deficit of specifically targeted body treatments. Our proposal for a body approach specifically dedicated to AN is based on the understanding that posture, breathing, muscle tension and body perception are closely linked to our psychological and emotional state and are therefore disturbed in patients with AN. The purpose of this monocentric randomized controlled trial is to evaluate if a targeted osteopathic protocol treatment for AN in addition to treatment as usual (TAU) is significantly more effective than TAU alone.

**Methods:**

In total, 72 patients meeting the inclusion criteria will be randomly assigned to one of the two treatment groups: one receiving the specific osteopathic treatment targeted for AN in addition to the TAU (group A) and the other one, receiving TAU only (group B). The patients in group A will receive 5 30-min osteopathic treatment sessions. Soft specific palpatory techniques on the diaphragm, digestive system and cervical region will be performed. The TAU is defined by the multidisciplinary approach recommended by the French health high authority. The primary outcome is the evaluation of interoceptive sensibility and secondary outcomes include clinical and psychopathology-related symptoms with assessment of somatic dysfunctions’ evolution. A qualitative study will also be carried out, applying the Interpretative Phenomenological Analysis method. Patients will be included for a maximum of 14 weeks between the inclusion time and the last evaluation.

**Discussion:**

If the results of the study are positive (statistically significant efficacy of this osteopathic treatment protocol), the study will provide arguments in favor of osteopathic sessions as a possible non-invasive additional treatment option in the multidisciplinary care approach for patients with AN.

**Trial registration:**

ClinicalTrials.gov ID: NCT04666415, Release Date: December 11, 2020; N° ID-RCB: 2019-A02613-54.

**Supplementary Information:**

The online version contains supplementary material available at 10.1186/s13063-021-05810-8.

## Background and rationale

Anorexia nervosa (AN) is defined by the DSM-5 as a restriction of energy relative to requirements, leading to a significantly low body weight, an intense fear of gaining weight or becoming fat, as well as disturbances in the way in which one’s body weight or shape is experienced and/or lack of recognition of the severity of current low body weight [1]. AN is a psychiatric disorder included among eating disorders (ED), with a reported prevalence in women ranging from 0.5 to 2.2% [2–4]. The age of onset of AN is mostly between 14 and 24 years old, and in half of the cases before the age of 18 [5]. Among all ED, AN is the one with the worst prognosis, with mortality 6 to 12 times that observed in a comparable general population of the same age [4], in particular due to somatic complications related to undernutrition, multiple hospitalizations, psychiatric comorbidities (mood, anxiety, personality and addictive disorders) and a significant suicidal risk [2, 4, 6–8]. According to main international guidelines, an overall assessment is recommended, combining a somatic, nutritional, and psychological assessment, also including social and family dynamics. An individualized care network has to be built for the patient and adjusted to his needs according to the evolution. It is a complex management, which requires a close collaboration between somaticians and child psychiatrists and, if possible, a dietician or a nutritionist [9–11].

AN perfectly illustrates the complex links between soma and psyche. It has been associated with profound alterations of body experiences and representations [12–14]. The perception of body image is wrong, distorted, sometimes almost delusional with a lack of knowledge or even a denial of thinness. Hilde Bruch was one of the first to identify these disturbances in body awareness, and to emphasize the absolute need to help patients become aware of their body’s perception and sensations [15]. Hypertonicity, body rigidity, and postural disorders are frequently associated with anorexic disorders. Muscular posture and tension are indeed closely related to the psychological and emotional state. Emotional avoidance leads to restricted breathing (not fully involving the balance between diaphragm and abdominal muscles), anxiety is the cause of chronic muscle tension, social withdrawal leads to body attitudes folded on oneself, and an impoverishment of spontaneous bodily movements, lack of self-confidence leads to poor postural stability [15].

The notion of embodied cognition (embodiment) suggests that the perception and treatment of an emotion involves its perceptive, motor, and somatovisceral experience [16, 17]. Interoceptive awareness describes behavioral, metacognitive, and cognitive processes related to internal bodily perceptions. Related to that concept, various studies showed behavioral interoceptive deficits in AN subjects with reduced interoceptive accuracy (that is, the ability to correctly perceive the internal sensations of the body) and also reduced interoceptive sensitivity (beliefs related to internal perceptions evaluated through questionnaires) [18, 19]. Alteration of the interoceptive consciousness could thus contribute to the symptoms of AN, for example the altered experience of hunger and satiety [20, 21], body image distortion, and alexithymia [18, 22].

Body-oriented care (relaxation, psychomotor therapy, aesthetic care, massage) is used in the care for patients suffering from AN [23]. The body approach and body image therapies allow adolescents to reinvest their body perceptions and sensations other than through deprivation and suffering and contribute to the improvement of their health status. It helps the adolescent break the body-mind division and the massive denial in which she/he is locked [23]. Unfortunately, studies on body-based approaches in AN are sparse and include small cohorts. They show little effect on weight evolution but a possible decrease in anxiety, frequent improvement of the thymia, and reduction in dietary concerns [24]. In addition, Adaptive Physical Activity Programs allow for progressive stress rehabilitation and have demonstrated beneficial action on the evolution of symptoms, particularly an improved self-perception [23].

There is a contrast between this consensual finding of the importance of body pattern disorders and body perception in AN, and the relative deficit of specifically targeted body care for AN. The body approach that we propose is based on the understanding that posture, breathing, muscle tension, and body perception are closely related to our psychological and emotional state, and therefore are disturbed in a patient suffering from AN. In osteopathic medicine, the osteopath, in a systemic approach after osteopathic diagnosis, carries out mobilizations and manipulations for the management of osteopathic dysfunctions of the human body.

To date, to our knowledge, there is no study based on the evaluation of an osteopathic treatment protocol in the management of patients with AN. Studies have focused on the contribution of osteopathy to intestinal functional disorders. Several studies have shown decreased abdominal pain in populations with chronic constipation [25] and irritable bowel syndrome [26, 27]. Another study showed improved abdominal pain and bloating, decreased use of medication, and improved quality of life for women with functional constipation after osteopathic treatment at a rate of one session per week for 1 month [28].

Thus, the ACAMTO study is exploratory research which aims to evaluate the impact of a targeted osteopathic treatment for AN within the framework of the recommended multidisciplinary approach for anorexic adolescent population.

## Objectives

### Primary objective

The main objective of this randomized parallel-group trial is to examine if the addition of a targeted osteopathic treatment to treatment as usual (TAU) for AN (multidisciplinary treatment program for adolescent with AN) is significantly more efficacious than TAU alone in terms of interoceptive sensitivity.

### Secondary objectives

Secondary objectives are as follows:

- To examine if adding a targeted osteopathic treatment to TAU for AN is significantly more efficacious than TAU alone in terms of clinical evolution, evaluated by other clinical outcomes (self-esteem, anxiety, depression, depressivity, alexithymia, quality of life, ED symptoms, physical hyperactivity, self-injury, body satisfaction, body mass index).
- To assess if adding a targeted osteopathic treatment to TAU for AN compared to TAU alone is significantly more efficacious in terms of the evolution of somatic dysfunctions (sensitivity, mobility, texture, asymmetry).
- To explore the subjective experience of patients who have completed osteopathic treatment sessions.
- To explore the subjective experience of osteopath students who have performed the osteopathic treatment sessions.

### Hypotheses

We developed, within the department of Psychiatry, a specific body approach for female adolescents hospitalized for AN. In collaboration with an experienced osteopath, YKM, we will offer an osteopathic treatment of 5 sessions performed over a week, in addition to the usual care offered to these patients. This approach is designed to enable anorexic teenage girls to improve their body perception and better embody their own body.

The hypothesis of this research is that a specific body approach to AN, based on osteopathic treatment sessions, in addition to the TAU, is significantly more efficacious than the TAU alone.

## Methods

### General design aspects

**Fig. 1:**
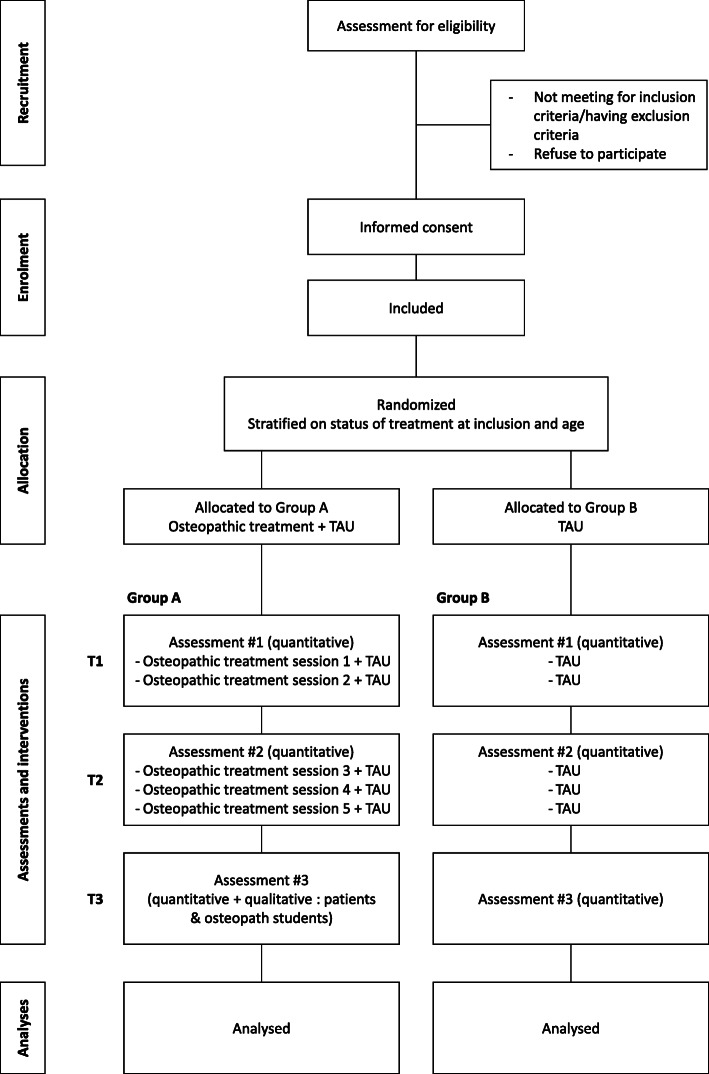
ACAMTO study participants flowchart. Legend: TAU = treatment as usual; T = time

This study is an exploratory add-on, monocentric, prospective, parallel-group, randomized controlled trial comparing a targeted osteopathic treatment in addition to TAU (multidisciplinary treatment program for adolescent with AN) versus TAU alone. Participants will be randomly allocated to either receive the 5 30-min sessions of osteopathic treatment spread over a week plus the TAU for AN (Group A) or receive the TAU alone (group B). Outcomes will be assessed at baseline, before the 3rd osteopathic session (group A) or 3 weeks after the first questionnaires (group B), and 1 week after the last osteopathic session (group A) or 6 weeks after the first questionnaires (group B). Patients will be included for a maximum of 14 weeks between the inclusion time and the last evaluation. The study protocol is outlined in the flowchart (Fig. 1).

The Standard Protocol items: Recommendations for Interventional Trials (SPIRIT) checklist is provided in Additional file 1.

### Participants

Patients will be recruited from the inpatient and outpatient units of the Adolescent and Young Adult Psychiatry department of the Institut Mutualiste Montsouris (Paris, France).

#### Inclusion criteria

All patients treated in outpatient care or hospitalized for AN in the psychiatry unit of the Institut Mutualiste Montsouris will be offered by the principal investigator or co-investigators to participate in the study if (1) they are female, (2) they are aged between 13 and 20 years old included, (3) they have a Diagnostic and Statistical Manual of Mental Disorders fifth edition (DSM-5) diagnosis of AN (restricting or binge-eating/purging types), (4) they have a body mass index (BMI) ≥ 14 kg m^−2^, (5) they have been treated in consultation for at least 2 months or have been hospitalized in the psychiatry unit of Institut Mutualiste Montsouris, (6) they have a clinical/somatic state compatible with osteopathy sessions (prior medical advice), (7) they are not in a “separation period”, (8) they have signed the informed consent form as well as both parents for patients under 18, and (9) they are not part of another research protocol.

At the Adolescent and Young Adult Psychiatry department of the Institut Mutualiste Montsouris, during a full-time hospitalization for AN, the “separation period” is linked to the establishment of a hospitalization contract between the medical team, the family, and the patient. This contract defines a time of separation between the family and the patient. It is built around two axes: the final discharge weight allowing the patient to leave the service and an intermediate weight called the “separation lifting weight.” This separation lifting weight is so named because it puts an end to the first part of the hospitalization which is carried out away from the living environment of the patient (parents, friends, schooling…).

#### Non-inclusion criteria

Patients will not be included in the study if (1) they are male, (2) they are under 13 years old or over 20 years old, (4) they have a contraindication to osteopathic body approach sessions (fracture, recent surgery, etc.), (5) they have a body mass index (BMI) < 14 kg m^−2^, (6) they need a naso-gastric tube, (7) they are in a “separation period”, (8) they are not fluent in French, (9) they are not covered by health insurance, and (10) they are part of another research protocol.

Non-included subjects will be listed along with the causes of their non-inclusion.

#### Exclusion criteria

Patients will be excluded from the research if (1) they have a contraindication to osteopathic body approach sessions (fracture, recent surgery, somatic state not compatible with the osteopathic treatment), (2) they are not covered by health insurance, and (3) they are part of another research protocol. Excluded subjects will be listed along with the causes of their exclusion.

#### Randomization

All patients who give consent to participate and who fulfill the inclusion criteria will be randomized. Randomization will be centralized and stratified according to the status of treatment at inclusion (outpatient care or hospitalization) and age (2 classes: 13–15 years old included and 16–20 years old). A randomization list with blocks of variable sizes will be computer-generated by a statistician independent from the group recruiting the patients and the group taking care of patients. The allocation ratio will be 1:1.

#### Blinding

Only osteopaths, their accompanying persons, and a psychologist coordinator (CDL) will receive information about group allocation, as well as patients and parents when the patient is under 18. The assessor of the clinical and psychological outcomes (research psychologist) will be blinded from the treatment group and will use standardized research questionnaires. Assessors of the osteopathic outcomes will not perform the osteopathic sessions, to guarantee an independent evaluation.

### Interventions

After their inclusion in the trial, the patients will receive either the targeted osteopathic treatment in addition to TAU (group A) or TAU alone (group B).

#### Osteopathic treatment (group A)

Special attention is paid to body approach care for anorexic patients in our psychiatry unit. A targeted osteopathic treatment for AN has been created by an osteopath (YKM), based on scientific literature and her own experience. The protocol was refined after 3 sessions for 3 different patients during the preparatory phase of the study. Indeed, in May 2019, 3 sessions of the osteopathic protocol were performed 1 week apart for 3 patients hospitalized in the department of psychiatry, after medical advice of no contraindication to the practice. The consent agreements of patients and their parents were collected and included in their medical records. Each intervention took place in full view of a caregiver of the unit as we usually do.

The osteopath, YKM, trained and supervised a group of osteopath students specially dedicated to the protocol, in their 3rd and 4th year of school.

Each patient allocated to group A will be followed by the same two osteopaths. One of them will provide the 5 sessions of osteopathic treatment. Sessions have a 30-min duration and about a week separates two sessions. The other one will evaluate the evolution of the somatic dysfunction. Two caregivers of the service will attend the sessions with the osteopaths and the patient. The presence of a caregiver during the therapeutic activities is customary in our unit.

The osteopathic treatment protocol will use techniques on 3 areas: cervical, diaphragmatic, and digestive. The protocol is detailed in Additional file 2.

#### TAU (for groups A and B)

The outpatient and inpatient treatment will be done in accordance with the French Health Authorities’ recommendations [11]. Multidisciplinary care is proposed, possibly involving nurses, psychologists, psychiatrists, general practitioners, gynecologists, rheumatologists, dieticians, occupational therapists, body approach specialists, and social workers. Targets for treatment include ED symptomatology, consequences of starvation, psychological disorders, and family interactions. A coordinating psychiatrist coordinates the care for AN. For a full description of inpatient and outpatient treatment modalities in our structure, see previous studies [29–31].

### Instruments

**Fig. 2:**
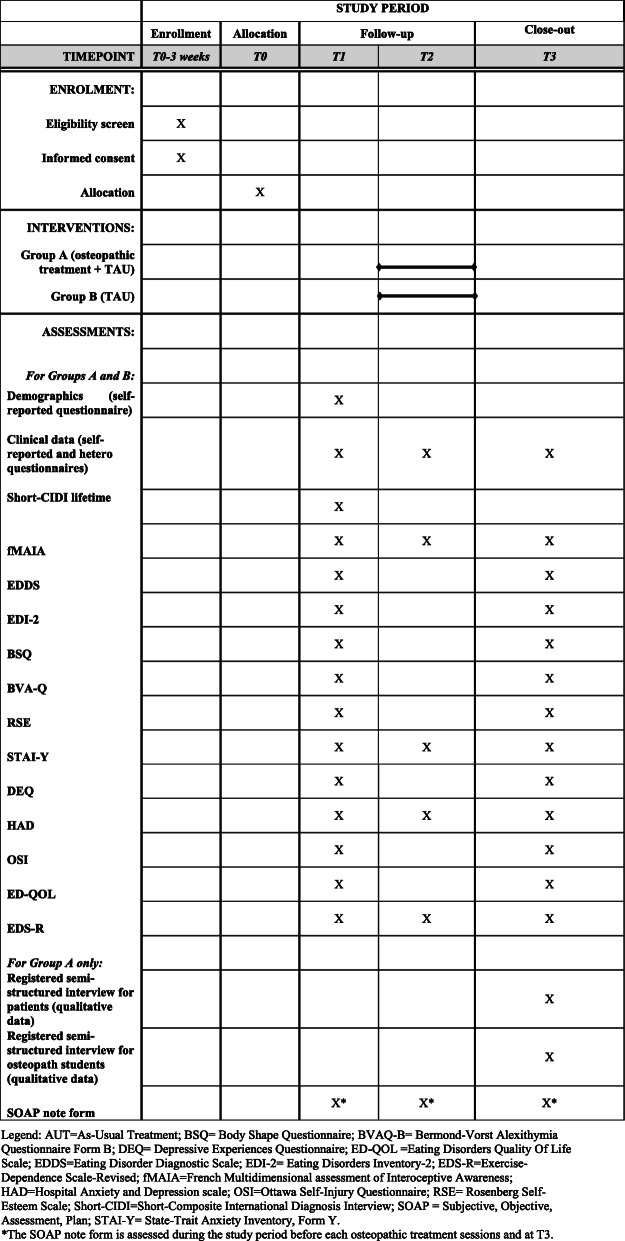
Schedule of enrolment, interventions and assessments for ACAMTO study (SPIRIT figure). Legend: AUT = as usual treatment; BSQ = Body Shape Questionnaire; BVAQ-B = Bermond-Vorst Alexithymia Questionnaire Form B; DEQ = Depressive Experiences Questionnaire; ED-QOL = Eating Disorders Quality Of Life Scale; EDDS = Eating Disorder Diagnostic Scale; EDI-2 = Eating Disorders Inventory-2; EDS-R = Exercise-Dependence Scale-Revised; fMAIA = French Multidimensional assessment of Interoceptive Awareness; HAD = Hospital Anxiety and Depression scale; OSI = Ottawa Self-Injury Questionnaire; RSE = Rosenberg Self-Esteem Scale; Short-CIDI = Short-Composite International Diagnosis Interview; SOAP = Subjective, Objective, Assessment, Plan; STAI-Y = State-Trait Anxiety Inventory, Form Y. *The SOAP note form is assessed during the study period before each osteopathic treatment sessions and at T3

Figure 2 summarizes the patients evaluations proposed at baseline, before the 3rd osteopathic session (group A) or 3 weeks after the first questionnaires (group B) and 1 week after the last osteopathic session (group A) or 6 weeks after the first questionnaires (group B).

Evaluations are described in the following paragraphs.

#### Clinical and demographic information

The patient’s demographic information will be collected through a self-report questionnaire, and clinical information (e.g., personal and family psychiatric history, history of illness, current treatment) will be collected through a semi-structured interview. Weight will be measured with the subject wearing only underwear on the same calibrated scales. Stature will be measured using a stadiometer.

#### Psychological quantitative evaluation

##### Short-CIDI

The Short-CIDI (Composite International Diagnostic Interview) form is a hetero-questionnaire diagnostic assessment instrument for the diagnosis of eating disorder (ED) [32]. The sections of the Short-CIDI relevant for the evaluation of ED will be used.

##### fMAIA scale (Multidimensional Assessment of Interoceptive Awareness, French version).

Interoceptive consciousness is a concept that describes behavioral, metacognitive, and cognitive processes related to internal bodily perceptions. The sub-components are as follows: interoceptive precision, metacognitive interoceptive consciousness, and interoceptive sensitivity [33]. The interoceptive sensitivity is concerned with the cognitive beliefs of the subjects in relation to their bodily perceptions. It is measured through certain scales such as MAIA (Multidimensional Assessment of Interoceptive Awareness) [34]. MAIA is a 32-item multidimensional scale with 8 sub-scales: (a) Noticing: awareness of uncomfortable, comfortable and neutral bodily sensations; (b) Not-Distracting: the tendency to not ignore or distract oneself from sensations of pain or discomfort; (c) Not-Worrying: the tendency to not react with emotional distress or worry to sensations of pain or discomfort; (d) Attention Regulation: the ability to sustain and control attention to bodily sensation; (e) Emotional Awareness: the awareness of the connection between bodily sensations and emotional states; (f) Self-Regulation: the ability to regulate psychological distress by attention to bodily sensations; (g) Body Listening: actively listening to the body for insight; and (h) Trusting: experiencing one’s body as safe and trustworthy. The scale was studied in an ED clinical population including restrictive AN, AN with purging behaviors, bulimia, bulimic hyperphagia, Avoidant Restrictive Food Intake Disorder (ARFID), and other specified EDs [35]. In this study, the dimensions of not-distracting, self-regulation, body listening, and trusting were more strongly associated with ED [35]. This scale was translated and validated in French, presented at the congress of the French Society of Psychology in 2016 [36]. The translated scale has good psychometric properties. The results show that the fMAIA follows a factor structure similar to that of the original version. The internal consistency is good (*α* = 0.83), as well as its stability over time.

##### Eating Disorder Diagnostic Scale (EDDS)

The Eating Disorder Diagnostic Scale (EDDS) is a self-administered questionnaire of 23 items used to assess the presence of AN, bulimia nervosa, and binge-eating disorder [37]. The scale is presented as a combination of questionnaires with Likert scales, yes/no scores, frequency scales, and open-ended questions.

##### Eating Disorders Inventory (EDI-2)

The Eating Disorders Inventory-2 (EDI-2) is intended to assess attitudes and behaviors related to eating habits [38, 39]. Although it is not diagnostic, it is often used in the context of a search for an ED to study the psychological characteristics of the subjects. Eleven dimensions are listed: drive for thinness, bulimia, body dissatisfaction, ineffectiveness, perfectionism, interpersonal distrust, interoceptive awareness, fear of maturity, asceticism, impulsivity regulation, and social insecurity. Each of the 91 items must be rated by the subject in six degrees ranging from “never” to “always.” Higher scores indicate higher level of symptoms.

##### Body Shape Questionnaire (BSQ)

The Body Shape Questionnaire (BSQ) is a one-dimensional self-assessment of 34 items used to assess preoccupations and concerns around body image, developed by Cooper and his colleagues and validated in French [40, 41]. The BSQ-34 scale includes four sub-scales: body dissatisfaction, fear of gaining weight, low self-esteem, and desire to lose weight. A high score indicates strong body image concern. The 34 items refer to the status of the last 4 weeks and the answers are in the form of a 6-point Likert scale from “never” to “always.” The total score ranges from 34 to 204. Scores below 80 indicate a lack of clinical significance of bodily concerns.

##### Bermond-Vorst Alexithymia Questionnaire Form B (BVAQ-B)

The Bermond-Vorst Alexithymia Questionnaire Form B (BVAQ-B) is a self-administered questionnaire that evaluates introspection and emotional regulation difficulties [42, 43]. This is a questionnaire of 20 items including 5 sub-scales: verbalization of emotional experiences (B1), waking dream and fantasies (B2), identification of emotions (B3), tendency to be awakened by events inducing emotions (B4), analysis of one’s own emotional states and reactions (B5). A high score indicates a high level of alexithymia.

##### Rosenberg Self-Esteem Scale (RSE)

The Rosenberg Self-Esteem Scale (RSE) is one of the most well-known and widely used scales in self-esteem assessment [44, 45]. This scale proposes ten items: five correspond to high self-esteem, and five correspond to low self-esteem. For each of the questions asked, the subject gives his appreciation on a Likert scale in four points ranging from “fully agree” (1) to “do not agree at all” (4). For positive questions, the score is added up. For negative questions, the rating is reversed. Possible scores range from 10 to 40, 40 representing the highest level of self-esteem, while 10 represents the lowest level of self-esteem. A score of 10 to 16 is in favor of low self-esteem, a score of 17 to 33 is an average self-esteem, and finally, a score of 34 to 40 shows high self-esteem.

##### State-Trait Anxiety Inventory, Form Y (STAI-Y)

The State-Trait Anxiety Inventory, Form Y (STAI-Y), described in 1983, is one of the most widely used self-assessment scales in research and clinical practice [46]. This scale was adapted in French and validated in 1990, then called STAI-Y [47]. The STAI-Y is a test to assess momentary anxiety and habitual anxiety. The STAI-Y consists of 2 scales of 20 items each. The State-Anxiety scale assesses the feelings of apprehension, tension, nervousness, and anxiety that the subject feels at the time of the consultation. It is an indicator of transient changes in anxiety caused by therapeutic or aversive situations. The Trait Anxiety Scale assesses feelings of apprehension, tension, nervousness, and anxiety that the subject usually feels. The purpose of this scale is to identify anxiety as a stable arrangement. Both scales can be used independently. Each item in the inventory is rated from 1 to 4, depending on its intensity. The total score thus varies from 20 to 80. For ease of interpretation, the results are classified into 5 levels: (1) very high stress (≥ 66); (2) high stress (56 to 65); (3) medium stress (46 to 55); (4) low stress (36 to 45); (5) very low stress (≤ 35).

##### Depressive Experiences Questionnaire (DEQ)—Adolescent Version

Blatt and colleagues have developed an original line of reflection on the primary sources of depression, based on both psychoanalytical and cognitive work [48]. It was validated in French with a teenage version [49]. Depression could be either correlated with feelings of abandonment and impotence, weakness, and exhaustion, or with feelings of self-esteem, self-criticism, shame, and guilt. The DEQ is a self-assessment of 66 items to assess “depressive experiences” and thus differentiate anaclitic from internalization-introjection depressions. This notion of “depressive experience” refers to the existence of a depressive tone or depressivity, which can be demonstrated before the onset of clinical symptoms of depression and the constitution of a major depressive disorder.

##### Hospital Anxiety and Depression (HAD) scale

The Hospital Anxiety and Depression (HAD) scale is a self-administered scale of 14 items [50, 51]. It contains 7 questions related to anxiety and 7 other questions related to the depressive dimension, allowing the obtention of 2 scores. Each item is rated on a scale from 0 to 3, for a total maximum score of 21 for each score. A score between 0 and 7 is considered normal, a score between 8 and 10 shows symptoms of moderate intensity, a score between 11 and 14 highlights symptoms of average intensity, and finally a score between 15 and 21 corresponds to a high intensity of symptoms.

##### Ottawa Self-injury Questionnaire (OSI)

The Ottawa Self-injury Questionnaire (OSI) is a self-assessment questionnaire of 12 questions, assessing the frequency and topicality of self-mutilation, the context of the onset of the behavior, the physical areas involved, and the motivations for this type of behavior [52].

##### Eating Disorders Quality of Life (ED-QOL) scale

The Eating Disorders Quality of Life (ED-QOL) scale is a self-questionnaire comprising 25 items and 4 sub-scales: psychological, physical/cognitive, work/school, and financial fields [53]. A higher score indicates a better quality of life.

##### Exercise-Dependence Scale-Revised (EDS-R)

The Exercise-Dependence Scale-Revised (EDS-R) scale is used to measure 7 dimensions through 21 items with a 6-point Likert scale [54, 55]. These seven dimensions are as follows: tolerance, weaning, lack of control, reduction of other activities, intention, time, and continuity.

##### Evaluations of osteopathic treatment with SOAP (Subjective, Objective, Assessment, Plan) note form

The SOAP (Subjective, Objective, Assessment, Plan) note form is a validated, standardized and easy-to-use tool for assessing the evolution of somatic dysfunctions (sensitivity, mobility, texture, asymmetry) [56].

This tool is recommended by professional osteopaths in the context of osteopathic research and studies.

### Outcomes

#### Primary outcome

The primary outcome is the evolution of interoceptive sensitivity using the fMAIA scale (Multidimensional Assessment of Interoceptive Awareness). We will calculate the difference between the fMAIA values between the last and the first evaluations.

#### Secondary outcomes

Secondary outcomes are as follows:

- Change in the patient’s overall clinical outcome (weight, pain, transit…) from T1 (baseline) to T3 (1 week after the last osteopathic session (group A) or 6 weeks after the first questionnaires (group B)).
- Change in the nature and severity of patient’s ED symptoms (EDDS and EDI-2) from T1 to T3.
- Change in the nature and severity of patient’s psychopathological symptoms (BSQ, BVAQ, RSE, STAI-Y, DEQ, HAD, OSI, EDS-R) from T1 to T3.
- Change in the perceived quality of life (ED-QOL) from T1 to T3.
- Change in somatic dysfunction (SOAP note form) from T1 to T3.
- Qualitative analysis of interviews of patients who received osteopathic treatment and osteopath students that performed the sessions (Interpretative Phenomenological Analysis method).

### Statistics

#### Sample size

To date, there is no quantitative data on which to base an order of expected effectiveness because this study is the first in the field. The calculation of the necessary number of subjects based on previous published research is therefore not possible. However, we carried out a clinical preparatory phase for the research by offering 3 osteopathy sessions of the initial treatment protocol to 3 patients with AN. They completed the MAIA questionnaire before and after the 3 sessions. For these 3 patients, total fMAIA values first and after evaluations were respectively 15.2 (sd = 7.9) and 23.2 (sd = 7.3) with an average difference of 7.97 (sd = 3.43). If the results of the study are positive, even if this sample size (*n* = 3) was too small to do statistical tests, that gave a favorable trend to support the study.

Finally, it seems essential that each of the two groups has more than 30 patients (use of parametric tests). Considering a 5 to 10% dropout rate, we calculated a sample size of 36 patients per treatment group.

In addition, this research is categorized as interventional research with only minimal risks and constraints mentioned 2 ° of Article L. 1121-2 of the Public Health Code. In this context, there are no anticipated formal stopping rules.

#### Statistical methods

##### Quantitative data

First, we will conduct descriptive analyses (calculations of means and standard deviation for the quantitative variables, percentages, and absolute numbers for the qualitative variables).

Quantitative variables will be analyzed with Student’s *t* test or a Mann-Whitney rank test if the validity conditions of the Student’s test are not validated. That will include the primary outcome as the difference between the fMAIA values between the last and the first evaluations, and the secondary outcomes such as the change in the patient’s clinical outcome (weight, pain…), the change in the nature and severity of patient’s ED symptoms (EDDS and EDI-2), the change in the nature and severity of patient’s psychopathological symptoms (BSQ, BVAQ, RSE, STAI-Y, DEQ, HAD, OSI, EDS-R), the change in the perceived quality of life (ED-QOL), and the change in somatic dysfunction (SOAP note form) from T1 to T3.

Qualitative variables will be analyzed with a chi-square test or an exact Fisher test if the validity conditions for chi-square are not validated. That will include the socio-demographic data and some secondary outcomes such as the change in the patient’s clinical outcome (localization of pain, transit…) from T1 to T3. Statistical tests will be two-sided with a significance level set at 0.05.

Relative to sample size, we did not plan any adjusted or additional analyses. In addition, we will collect different information for each patient at each stage of the protocol (inclusion, randomization, assessments). Subjects randomized to a group that will ultimately do not join their randomization group will be out of the study. We have estimated for the calculation of the sample size a dropout rate of 5 to 10%, which remains compatible with sufficient statistical power. We will compare the characteristics of dropout subjects compared to those who completed the study to investigate possible biases.

##### Qualitative data

We will collect the individual stories of patients who received osteopathic treatment from a semi-structured interview grid, which will be recorded (date, file number of research, and interview) on a voice recorder in order to allow a written transcription. At the end of the last osteopathic treatment session of a patient in Group A, we will also offer the osteopathic students to participate in a qualitative interview.

Throughout the interview, the subject is encouraged to speak freely, associate, and develop ideas. The guide remains an indicative framework to promote the narrative process. It will be modified according to the course of the interview to fluidify the exchange and not hinder the collection of data. The participant is the expert of his own experience.

Data analysis will be based on the IPA (Interpretative Phenomenological Analysis) method, a classical method for exploiting narratives. The IPA is more specifically centered on the personal meaning of the person’s experiences. The IPA is thus a “qualitative approach to research engaged in examining how people give meaning to their main life experiences”. Its approach is basically inductive and interpretive. It is phenomenological insofar as it explores experience with its own terms. It is hermeneutic to the extent that it is a “theory of interpretation.”

A double rating of the data is done to increase the rigor of the analysis. The identity of each of the researchers will be specified.

The semi-structured interview questions are as follows:

For patients who have completed the osteopathic sessions to explore the subjective experience of these sessions:

- Can you describe an osteopathy session? (Alternative: How does an osteopathy session go?)
- How did you feel during the sessions? How did you feel right after? What did you like the most/least? (Alternatives: Did you find this useful? Have you ever felt bad during or after a session? What could you say about your perception of your own body? of your body image?)
- What did the osteopathic sessions bring for you outside of the sessions? (Alternatives: did you see any benefit between sessions? Movement, breathing, tone…? When? How?)
- What does osteopathy mean to you? (Alternatives: What do you think? Does it make sense?)
- Have you talked about your experience of osteopathy around you? (Alternatives: outside sessions? with your family? psychiatrists? nursing team? other patients?)
- Compared to other activities offered in the management of your anorexia nervosa, what is the specificity of osteopathy sessions for you?
- Would you like to continue osteopathic treatment after this proposed treatment?
- Could you summarize your opinion on osteopathy sessions?

For osteopath students who have practiced osteopathic treatment sessions to explore their subjective experience of the sessions, their work, and meeting with each patient:

- Can you describe the evolution of the patient through osteopathic treatment? (Alternatives: what did you think of the patient’s management? What changed, what was modified for this patient?)
- How did you feel during the sessions? What do you feel at the end of this treatment? (Alternative: did you feel any difficulty during this treatment?)

### Data collection and management

Data will be transferred to observation paper files as it is collected, whether it is clinical or para-clinical data (including possible adverse events). The psychologist coordinator will collect the data from paper files (anonymous Case Report Form with patient codes) and will entry the data in an Excel document. The list of correspondence between patient codes and clear names and surnames shall be kept under the responsibility of the principal investigator in a locked cabinet and will be only known by the psychologist coordinator. The statistician will give the result of randomization to the research coordinator depending on the necessary data for stratification. The research coordinator will enter the result of the randomization on the paper form. This circuit is independent of the principal investigator and associate investigators. A clinical research assistant appointed by the trial sponsor will keep the database on the hospital server with daily back-up on an outsourced server. For the data quality control, a double manual quality-check of the paper files will be carried out by the research psychologist and the psychologist coordinator who is used to coordinate research for many years in our department. Random checks will be made between Excel and paper data after entry. Missing and erroneous data (e.g., out-of-range data) will be detected by the statistician and reported to the investigator for correction. Randomization program and data management procedures will be stored as R program code files and will be accessible, as well as anonymous data used for randomization.

Essential research documents that come under the law on biomedical research will be archived by all parties for 15 years after the end of research. This trial does not involve collecting biological specimens for storage. To ensure confidentiality, the access to the trial data will be limited to the minimum number of necessary individuals, e.g., the trial team and sponsor organization. Each patient will have an individual trial identification number, his personal data is therefore collected in complete security. The anonymized trial data will not be share with other researchers to enable international prospective meta-analyses.

The trial is registered with the French National Commission for Data Protection and Liberties (CNIL) under chapter IX of law no. 78-17 of 6 January 1978, modified by law no. 2016-41 of 26 January 2016, regarding the treatment of personal data for the purpose of scientific research in the health field. The investigator and the members of his team agree to make themselves available during the Quality Control visits carried out at regular intervals by the Clinical Research Assistant mandated by the sponsor.

The research is categorized as interventional research with only minimal risks and constraints mentioned 2 ° of Article L. 1121-2 of the Public Health Code. Thus, there is no vigilance circuit apart from the one we use in daily care. Osteopaths have an internship agreement and insurance. There is no anticipated harm and compensation for trial participation, and no provision for post-trial care. At the end of the trial, all the subjects will go on their usual treatment.

Any modification from the protocol will be fully documented with a new written document. The sponsor and the foundation will be informed of the changes. We will update the protocol in the clinical trial registry.

## Discussion

The scientific literature highlights body-related disorders in AN, either in terms of perceptions, experiences, or body representations. Offering body-mediated care seems to be particularly indicated. However, despite the idea that body approach techniques may be interesting in AN, there is a relative deficit in care proposals specifically dedicated to this disorder. The ACAMTO study is exploratory research that aims to evaluate the impact of a targeted osteopathic treatment for AN within the framework of the recommended multidisciplinary approach for the anorexic adolescent population. The main objective of this study is to examine if the addition of a targeted osteopathic treatment to TAU (multidisciplinary treatment program for adolescent with AN) for AN is significantly more efficacious than TAU in terms of interoceptive sensitivity. Secondary objectives are to examine if adding a targeted osteopathic treatment to TAU for AN compared to TAU alone is significantly more efficacious in terms of clinical evolution, evaluated by other clinical and psychopathological outcomes (self-esteem, anxiety, depression, depressivity, alexithymia, quality of life, ED symptoms, physical hyperactivity, self-injury, body dissatisfaction, body mass index, somatic dysfunctions).

From our point of view, our study presents important strengths. First, to our knowledge, this is the first study to evaluate the impact of an osteopathic treatment on adolescent patients with AN. The protocol was specifically studied and developed for patients with AN, meaning the techniques and the areas of treatment were specifically adapted for them. It enhances its individualized and personalized specificities. Secondly, the osteopathic treatment protocol has been standardized by an experienced osteopath (YKM). And the interest of a standardized protocol is to propose an evidence-based and reproducible treatment. Thirdly, a special feature of the study design is an add-on trial, meant to study the impact of osteopathic management in addition to the multidisciplinary care already offered. If the results of the study show an efficacy of osteopathic techniques in addition to the TAU, this will allow us to improve therapeutic indications and achieve greater and earlier efficacy.

The trial also faces challenges that we have to consider. Several potential practical issues may challenge the successful and timely completion of the study, particularly regarding the recruitment and attrition. Indeed, we have to recruit 72 patients with DSM-5 AN criteria while the reported prevalence in women ranges from 0.5 to 2.2% [2–4]. However, the recruitment is done in a department with a specialization for ED including AN, and the projections based on the regular active list of patients in consultation and hospitalization units make us confident about an effective recruitment in the dedicated time. In addition, patients with ED are known to be ambivalent about the treatment and to suffer from denial. That can be difficult if the treatment or the assessment are experienced as too burdensome. The psychologists in charge of coordinating the study and evaluations are aware of this issue and have already experienced the management of follow-up evaluations of patients with eating disorders.

Finally, another challenge concerns minimization of bias linked to osteopath students. Indeed, all osteopath students involved in the trial have been trained by YKM who developed the protocol. We expect to minimize the potential bias by regular training and regular communication between the students and the referent osteopath (YKM).

To conclude, if the results of the study are positive (statistically significant efficacy of this osteopathic treatment protocol), the study will provide arguments in favor of osteopathic sessions as a possible non-invasive additional treatment option in the multidisciplinary care approach for patients with AN. We have to remember here that no drug, psychotropic or non-psychotropic, has a marketing authorization—in France or worldwide—specially dedicated to AN.

Considering the severe prognosis of AN, the difficulties encountered in its management, the expected benefits in terms of Public Health are important.

## Trial status

The trial was registered on ClinicalTrials.gov ID: NCT04666415, Release Date: December 11, 2020 (https://clinicaltrials.gov/ct2/show/NCT04666415); IDRCB number: 2019-A02613-54. Recruitment for the trial started in February 2020 but was stopped because of the COVID-19 health crisis in March 2020 and started again in October 2020. We scheduled an end of recruitment in April 2022. Participant recruitment and data collection have already begun at the time of manuscript submission.

## Supplementary Information

**Additional file 1.**

**Additional file 2.**

## Data Availability

The Institut Mutualiste Montsouris is the owner of the data of the research. The Institut Mutualiste Montsouris is the Sponsor of the study. Contact details: Institut Mutualiste Montsouris- regulatory: Clinical Research, Quality and Risks Direction, phone number: + 33 1 56 61 67 05.

## Abbreviations

AN: Anorexia nervosa
BMI: Body mass index
ED: Eating disorder

## References

[1] American Psychiatric Association (2013). Diagnostic and statistical manual of mental disorders.

[2] Hudson JI, Hiripi E, Pope HG, Kessler RC. The prevalence and correlates of eating disorders in the National Comorbidity Survey Replication. Biol Psychiatry. 1 févr 2007;61(3):348-358. 10.1016/j.biopsych.2006.03.040 PMC1892232 16815322

[3] Keski-Rahkonen A, Hoek HW, Susser ES, Linna MS, Sihvola E, Raevuori A (2007). Epidemiology and course of anorexia nervosa in the community. Am J Psychiatry. août.

[4] Roux H, Chapelon E, Godart N. [Epidemiology of anorexia nervosa: a review]. Encephale. avr 2013;39(2):85-93. 10.1016/j.encep.2012.06.001 23095584

[5] Nagl M, Jacobi C, Paul M, Beesdo-Baum K, Höfler M, Lieb R, Wittchen HU (2016). Prevalence, incidence, and natural course of anorexia and bulimia nervosa among adolescents and young adults. Eur Child Adolesc Psychiatry. août.

[6] Berkman ND, Lohr KN, Bulik CM (2007). Outcomes of eating disorders: a systematic review of the literature. Int J Eat Disord. mai.

[7] Godart NT, Perdereau F, Rein Z, Berthoz S, Wallier J, Jeammet P, Flament MF (2007). Comorbidity studies of eating disorders and mood disorders. Critical review of the literature. J Affect Disord. janv.

[8] Baker JH, Mitchell KS, Neale MC, Kendler KS. Eating disorder symptomatology and substance use disorders: prevalence and shared risk in a population based twin sample. Int J Eat Disord. 1 nov 2010;43(7):648-658. 10.1002/eat.20856 PMC2972646 20734312

[9] National Collaborating Centre for Mental Health (UK). Eating disorders: core interventions in the treatment and management of anorexia nervosa, bulimia nervosa and related eating disorders. Leicester (UK): British Psychological Society (UK); 2004. PMID: 23346610. 23346610

[10] Yager J, Devlin MJ, Halmi K, Herzog DB, III JE, Powers P, et al. Practice guideline for the treatment of patients with eating disorders third edition. American Journal of Psychiatry. 1 juill 2006;163:1-128.

[11] Anorexie mentale : prise en charge. Recommandations juin 2010. Journal de pédiatrie et de puériculture 2012;25:00–47.

[12] Cash TF, Deagle EA (1997). The nature and extent of body-image disturbances in anorexia nervosa and bulimia nervosa: a meta-analysis. Int J Eat Disord. sept.

[13] Dalhoff AW, Romero Frausto H, Romer G, Wessing I. Perceptive body image distortion in adolescent anorexia nervosa: changes after treatment. Front Psychiatry. 2019 Oct 15;10:748. doi: 10.3389/fpsyt.2019.00748. PMID: 31681048; PMCID: PMC6803517. 10.3389/fpsyt.2019.00748 PMC6803517 31681048

[14] Di Lernia D, Serino S, Polli N, Cacciatore C, Persani L, Riva G. Interoceptive axes dissociation in anorexia nervosa: a single case study with follow up post-recovery assessment. Front Psychol. 2019 Jan 17;9:2488. doi: 10.3389/fpsyg.2018.02488. PMID: 30705649; PMCID: PMC6345152. 10.3389/fpsyg.2018.02488 PMC6345152 30705649

[15] Kolnes L-J (2012). Embodying the body in anorexia nervosa--a physiotherapeutic approach. J Bodyw Mov Ther. juill.

[16] Niedenthal PM, Winkielman P, Mondillon L, Vermeulen N (2009). Embodiment of emotion concepts. Journal of Personality and Social Psychology..

[17] Rossignol M, Philippot P, Vögele C. Réactivité physiologique et conscience intéroceptive dans les troubles anxieux pédiatriques : une revue conceptuelle et empirique [Physiological reactivity and interoceptive awareness in pediatric anxiety disorders: A conceptual and empirical review]. Sante Ment Que. 2016 Spring;41(1):183-222. French. PMID: 27570957. 27570957

[18] Pollatos O, Kurz A-L, Albrecht J, Schreder T, Kleemann AM, Schöpf V, Kopietz R, Wiesmann M, Schandry R (2008). Reduced perception of bodily signals in anorexia nervosa. Eat Behav. déc.

[19] Fischer D, Berberich G, Zaudig M, Krauseneck T, Weiss S, Pollatos O. Interoceptive processes in anorexia nervosa in the time course of cognitive-behavioral therapy: a pilot study. Front Psychiatry. 2016 Dec 15;7:199. doi: 10.3389/fpsyt.2016.00199. PMID: 28018249; PMCID: PMC5156660. 10.3389/fpsyt.2016.00199 PMC5156660 28018249

[20] Herbert BM, Blechert J, Hautzinger M, Matthias E, Herbert C (2013). Intuitive eating is associated with interoceptive sensitivity. Effects on body mass index. Appetite. nov.

[21] Brown TA, Berner LA, Jones MD, Reilly EE, Cusack A, Anderson LK, Kaye WH, Wierenga CE Psychometric evaluation and norms for the Multidimensional Assessment of Interoceptive Awareness (MAIA) in a Clinical Eating Disorders Sample. Eur Eat Disord Rev. sept 2017;25(5):411-416, DOI: 10.1002/erv.2532. 10.1002/erv.2532 28714581

[22] Kerr KL, Moseman SE, Avery JA, Bodurka J, Zucker NL, Simmons WK. Altered insula activity during visceral interoception in weight-restored patients with anorexia nervosa. Neuropsychopharmacology. janv 2016;41(2):521-528, DOI: 10.1038/npp.2015.174. 10.1038/npp.2015.174 PMC5130127 26084229

[23] Blanchet-Collet C, Moro MR. Anorexie mentale, soigner et prendre soin du corps maltraité. Soins Pédiatrie/Puériculturex. janv 2015;36(286):17-21, DOI: 10.1016/j.spp.2015.07.003. 10.1016/j.spp.2015.07.003 26381067

[24] Moscone AL, Leconte P, Scanff CL (2011). Perception de soi et activité physique adaptée dans l’anorexie mentale. Science & Sports..

[25] Modlin SE, Borofka K, Franzini D, Klene-Bowns AC, Nuño VA (2019). OMT for the prevention and management of chronic constipation and distal intestinal obstructive syndrome in cystic fibrosis: a pilot study. J Am Osteopath Assoc.

[26] Müller A, Franke H, Resch KL, Fryer G. Effectiveness of osteopathic manipulative therapy for managing symptoms of irritable bowel syndrome: a systematic review. J Am Osteopath Assoc 2014;114(6):470-479. doi: 10.7556/jaoa.2014.098. PMID: 24917634. 10.7556/jaoa.2014.098 24917634

[27] Attali TV, Bouchoucha M, Benamouzig R. Treatment of refractory irritable bowel syndrome with visceral osteopathy: short-term and long-term results of a randomized trial. J Dig Dis. 2013;14(12):654-661. doi: 10.1111/1751-2980.12098. PMID: 23981319. 10.1111/1751-2980.12098 23981319

[28] Belvaux A, Bouchoucha M, Benamouzig R. Osteopathic management of chronic constipation in women patients. Results of a pilot study. Clin Res Hepatol Gastroenterol. janv 2017;41(5):602-611, DOI: 10.1016/j.clinre.2016.12.003. 10.1016/j.clinre.2016.12.003 28215390

[29] Godart N, Atger F, Perdereau F, Agman G, Rein Z, Corcos M, Jeammet P. Treatment of adolescent patients with eating disorders: description of a psychodynamic approach in clinical practice. Eat Weight Disord. 1 sept 2004;9(3):224-227. 10.1007/BF03325071 15656018

[30] Godart N, Perdereau F, Galès O, Agman G, Deborde A-S, Jeammet P (2005). The weight contract during the hospitalization of anorexic patients. Arch Pediatr.

[31] Godart N, Wallier J, Hubert T, Curt F, Galès O, Perdereau F, Agman G, Jeammet P (2009). Detemining factors for target weights in an anorexia nervosa inpatient program for adolescents and young adults: study on the links between theory-based hypotheses and the realities of clinical practice. Eat Weight Disord.

[32] Kessler RC, Andrews G, Mroczek D, Ustun B, Wittchen H-U (1998). The World Health Organization Composite International Diagnostic Interview short-form (CIDI-SF). International Journal of Methods in Psychiatric Research..

[33] Garfinkel SN, Seth AK, Barrett AB, Suzuki K, Critchley HD (2015). Knowing your own heart: distinguishing interoceptive accuracy from interoceptive awareness. Biol Psychol. janv.

[34] Mehling WE, Price C, Daubenmier JJ, Acree M, Bartmess E, Stewart A. The Multidimensional Assessment of Interoceptive Awareness (MAIA). PLOS ONE. 1 nov 2012;7(11):e48230. 10.1371/journal.pone.0048230 PMC3486814 23133619

[35] Brown TA, Berner LA, Jones MD, Reilly EE, Cusack A, Anderson LK, Kaye WH, Wierenga CE (2017). Psychometric evaluation and norms for the Multidimensional Assessment of Interoceptive Awareness (MAIA) in a Clinical Eating Disorders Sample. Eur Eat Disord Rev. sept.

[36] Michael GA, Magnin C, Hamon J, Longo L, Blancel V, Echalier A, Borg C. Adaptation Française de la Multidimensional Assessment of Interoceptive Awareness (fMAIA) [French Adaptation of the Multidimensional Assessment of Interoceptive Awareness (fMAIA)]. Paper presented at: 57ème Congrès de la Société Française de Psychologie; September 7–9, 2016; Strasbourg. French.

[37] Stice E, Telch CF, Rizvi SL (2000). Development and validation of the Eating Disorder Diagnostic Scale: a brief self-report measure of anorexia, bulimia, and binge-eating disorder. Psychol Assess. juin.

[38] Garner DM, Olmstead MP, Polivy J (1983). Development and validation of a multidimensional eating disorder inventory for anorexia nervosa and bulimia. International Journal of Eating Disorders..

[39] Lavoisy G, Guelfi JD, Vera L, Dardennes R, Rouillon F. Evaluation des préoccupations corporelles dans les troubles des conduites alimentaires par le Body Shape Questionnaire [Evaluation of perturbed body image in eating disorders using the Body Shape Questionnaire]. Encephale. 2008 Dec;34(6):570-6. French. doi: 10.1016/j.encep.2007.11.005. Epub 2008 Apr 2. PMID: 19081453. 10.1016/j.encep.2007.11.005 19081453

[40] Cooper PJ, Taylor MJ, Cooper Z, Fairbum CG. The development and validation of the body shape questionnaire. International Journal of Eating Disorders. 1 janv 1987;6:485-94.

[41] Rousseau A, Knotter A, Barbe P, Raich R, Chabrol H. [Validation of the French version of the Body Shape Questionnaire]. Encephale. avr 2005;31(2):162-73. 10.1016/s0013-7006(05)82383-8 15959443

[42] Deborde A-S, Berthoz S, Perdereau F, Godart N, Corcos M, Jeammet P. [Validity of the BVAQ: a study in eating disorder patients and controls]. Encephale. oct 2004;30(5):464-73. 10.1016/s0013-7006(04)95461-9 15627051

[43] Vorst HCM, Bermond B. Validity and reliability of the Bermond–Vorst Alexithymia Questionnaire. Personality and Individual Differences. 1 févr 2001;30(3):413-34.

[44] Petersen W. Society and the Adolescent Self-Image. Morris Rosenberg. Princeton University Press, Princeton, N.J., 1965. Science. 7 mai 1965;148(3671):804-804.

[45] Vallieres EF, Vallerand RJ (1990). Traduction Et Validation Canadienne-Française De L’échelle De L’estime De Soi De Rosenberg*. International Journal of Psychology..

[46] Spielberger CD, Gorsuch RL, Lushene RE (1968). State-trait anxiety inventory (STAI): test manual for form X.

[47] Martin JD, Blair GE, Hatzel DJ. Rorschach correlates of state and trait anxiety in college students. Percept Mot Skills. 1987;64(2):539-543. doi: 10.2466/pms.1987.64.2.539. PMID: 3588196. 10.2466/pms.1987.64.2.539 3588196

[48] Blatt SJ, D’Afflitti JP, Quinlan DM (1976). Experiences of depression in normal young adults. J Abnorm Psychol. août.

[49] Atger F, Frasson G, Loas G, Guibourgé S, Corcos M, Perez Diaz F, Speranza M, Venisse JL, Lang F, Stephan P, Bizouard P, Flament M, Jeammet P. Etude de validation du Depressive Experience Questionnaire [Validation study of the Depressive Experience Questionnaire]. Encephale. 2003 Sep-Oct;29(5):445-55. French. PMID: 14615694. 14615694

[50] Lépine JP, Godchau M, Brun P, Lempérière T. Evaluation de l'anxiété et de la dépression chez des patients hospitalisés dans un service de médecine interne [Evaluation of anxiety and depression among patients hospitalized on an internal medicine service]. Ann Med Psychol (Paris). 1985 Feb;143(2):175-89. French. PMID: 4037594. 4037594

[51] Zigmond AS, Snaith RP (1983). The hospital anxiety and depression scale. Acta Psychiatr Scand. juin.

[52] Nixon MK, Levesque C, Preyde M, Vanderkooy J, Cloutier PF. The Ottawa Self-Injury Inventory: evaluation of an assessment measure of nonsuicidal self-injury in an inpatient sample of adolescents. Child Adolesc Psychiatry Ment Health. 2015 Jul 8;9:26. doi: 10.1186/s13034-015-0056-5. PMID: 26157482; PMCID: PMC4495629. 10.1186/s13034-015-0056-5 PMC4495629 26157482

[53] Engel SG, Wittrock DA, Crosby RD, Wonderlich SA, Mitchell JE, Kolotkin RL (2006). Development and psychometric validation of an eating disorder-specific health-related quality of life instrument. Int J Eat Disord. janv.

[54] Downs DS, Hausenblas HA, Nigg CR. Factorial Validity and Psychometric Examination of the Exercise Dependence Scale-Revised. Measurement in Physical Education and Exercise Science. 1 déc 2004;8(4):183-201.

[55] Kern L. Validation de l’adaptation française de l’échelle de dépendance à l’exercice physique: l’EDS-R. Pratiques Psychologiques. 1 déc 2007;13(4):425-41.

[56] Sleszynski SL, Glonek T. Outpatient Osteopathic SOAP Note Form: preliminary results in osteopathic outcomes-based research. J Am Osteopath Assoc. 2005;105(4):181-205. PMID: 15928337. 15928337

