# Supplementary material for: The ACAMTO study—impact of add-on osteopathic treatment on adolescent patients with anorexia nervosa: study protocol for a randomized controlled trial
Source: Trials. 2021 Nov 24;22:839. doi: 10.1186/s13063-021-05810-8 (PMC8611636; doi:10.1186/s13063-021-05810-8)
Supplement: Supplementary file 2 — Additional file 2. [file 13063_2021_5810_MOESM2_ESM.docx]

Osteopathic treatment protocol

1. Osteopathic techniques on the cervical area

**Objectives of cervical osteopathic treatment:** action on the vagus nerve at the level of its passage in the jugular foramen

**Role of the vagus nerve:** Gastric contraction; Release of the pylorus; Stimulation of secretion; Application of the osteopathic technique on the cervical region

*Global cervical release*

| **Position of the patient** | Supine position |
| --- | --- |
| **Position of the practitioner** | standing at the head of the patient with both feet in a split |
| **Palpation parameter** | Slide the four outstretched fingers overlaid with both hands under the high dorsal vertebrae, elbows outstretched, leaning on the front foot |
| **Technical aspects** | Reassemble along the neck, making sure not to compress the carotids and jugulars, avoiding cervical extension  Lift the patient’s head up  Perform a cephalic traction, pressing the rear foot  Pose and release the head  Repeat 2 – 3 times |

*Loosening of the sub-occipital muscles*

| **Position of the patient** | Supine position |
| --- | --- |
| **Position of the practitioner** | Sitting at the head of the patient, feet on the ground, knees apart, forearms horizontal, placed on the edge of the tilting table, elbows out |
| **Palpation parameter** | the four fingers of both hands in contact with the occipital curved line, auricular in contact with the inion |
| **Technical aspects** | Bend your fingers without grabbing, lower your elbows and carry the trunk slightly back, back straight  Hold until release  Move fingers sideways, once or twice |

*Release of the temporo-mandibular joint*

| **Position of the patient** | Supine position |
| --- | --- |
| **Position of the practitioner** | Seated at the head of the patient, forearms horizontal |
| **Palpation parameter** | palms of the hand on the gonions, fingers under the floor of the mouth |
| **Technical aspects** | Ask the patient to perform a straight diduction for 3 seconds against the resistance and then release  Perform 3 times and repeat ~~left~~on the other side |

2. Osteopathic Techniques on the diaphragm

*Description of diaphragmatic pillar release*

| **Position of the patient** | Supine position, legs bent, feet on the table |
| --- | --- |
| **Position of the practitioner** | standing on the side to be treated |
| **Palpation parameter** | One hand under spinous processes, crochet with index fingers at T12, major at L1, ring at L2 and auricular at L3  The other hand is placed on the rib cage in the intercostal space between K7 to K12 with the thumb in the diaphragmatic dome |
| **Technical aspects** | Ask the patient to exhale and apply slight pressure with the anterior hand. The posterior hand fixes the spinous processes to avoid lumbar rotation.  Maintain pressure until tissue release  Perform the same technique on the other side  Attention: the right diaphragmatic pillar goes down to L3 and the left to L2 |

3. Visceral osteopathic techniques for sphincters and digestive tract

*Release of the sigmoid*

| **Position of the patient** | Supine position, legs bent, feet on the table |
| --- | --- |
| **Position of the practitioner** | standing, to the right side of the patient |
| **Palpation parameter** | the practitioner makes contact with the lower part of the sigmoid colon in the left iliac fossa |
| **Technical aspects** | Practitioner pulls sigmoid colon to patient’s right shoulder  Then towards the head (cephalic traction) |

*Release of the sphincter of Oddi*

| **Position of the patient** | Supine position, legs bent, feet on the table |
| --- | --- |
| **Position of the practitioner** | standing to the right side of the patient, facing the patient |
| **Palpation parameter** | the practitioner places his right hand flat next to D2 and tightens his right wrist with his left hand to penetrate during the exhalation |
| **Technical aspects** | While maintaining pressure, the practitioner performs a clockwise rotation, at the maximum of the hourly rotation, maintaining power for 5 to 7 seconds.  During an inspiratory phase, he lets the tissues return to their initial position, then he performs an anti-clockwise rotation until release |

*Release of the pylorus*

| **Position of the patient** | Supine position, bent legs, feet on the table |
| --- | --- |
| **Position of the practitioner** | standing, to the right side of the patient |
| **Palpation parameter** | pisiform in contact with the pylorus |
| **Technical aspects** | The practitioner performs a clockwise rotational motion, maximizing power up, while maintaining the same pressure  Maintain tension for a few seconds until release |
